# A Bibliometric Analysis of Strabismus Research Literature: Strabismus Research Trends

**DOI:** 10.31661/gmj.vi.3887

**Published:** 2025-08-04

**Authors:** Minglian Ye, Jianzhong Yang, Jiamei Luo

**Affiliations:** ^1^ ABST Quanzhou First Hospital Affiliated to Fujian Medical University, Quanzhou 362000, Fujian, China

**Keywords:** Bibliometric Analysis, Intermittent Exotropia (IXT), Strabismus, Strabismus Surgery, Visual Axis Alignment

## Abstract

**Background:**

Background: Strabismus, a common ocular disorder marked by misalignment of the visual axes, can impair depth perception and visual function, while also affecting facial appearance and psychosocial wellbeing. In recent years, the field has seen a growing body of research focusing on its pathogenesis, risk factors, and therapeutic approaches. However, the literature is still fragmented, making it difficult to assess overarching trends. Therefore, a comprehensive bibliometric analysis is needed to understand research developments and identify emerging hotspots in this domain.

**Materials and Methods:**

Materials and Methods: A bibliometric analysis was conducted using VOSviewer and CiteSpace on 6,540 English-language articles and reviews related to strabismus, published between 1995 and 2025, and retrieved from the Web of Science Core Collection (WOSCC).

**Results:**

Results: The updated analysis revealed consistent growth in publication output, with particularly rapid expansion in recent years. While the United States remained a key contributor, China has overtaken the U.S. in annual output since 2023. Keyword co-occurrence and burst analysis identified both long-standing research interests (e.g., intermittent exotropia [IXT], AACE, surgical outcomes) and newer focus areas such as digital screen exposure, artificial intelligence in diagnostics, and individualized surgical planning.

**Conclusion:**

Conclusion: This study provides an updated and comprehensive bibliometric evaluation of global strabismus research through 2025. The results highlight evolving academic contributions, changing geographical trends, and shifting research priorities, offering guidance for future investigations and clinical advancements.

## Introduction

Strabismus encompasses a wide range of conditions characterized by misalignment of the eyes, disrupting the coordinated movement of the extraocular muscles. This disorder not only affects appearance but also leads to amblyopia, a loss of stereopsis, and a range of psychosocial issues, significantly diminishing the quality of life. The global prevalence of strabismus is estimated at 1.93%, with its occurrence varying across different populations and over time [1]. It can arise as a result of systemic conditions or be secondary to damage to the nerves, muscles, or orbital tissues, but the majority of cases are primary, particularly in children, with approximately 5% of normally developing children affected by the condition [2].

Although the diagnostic criteria for strabismus, primarily based on the alternate cover test, are well-established, the underlying mechanisms remain inadequately understood [3]. There is limited exploration into the factors contributing to its development, including genetic mutations, refractive status, binocular vision, and control from the visual center [4][5][6][7]. Treatment, while primarily surgical, faces challenges due to individual variability in responses. The success rates for surgical interventions in basic horizontal comitant strabismus are reported to be less than 60% in long-term follow-ups [8][9][10]. Surgery for more complex cases is particularly difficult due to the lack of a clear dose-effect relationship [11]. Furthermore, non-surgical treatments show inconsistent results [12]. To advance strabismus research, a comprehensive review of the latest publications is essential. Bibliometric analysis offers a robust approach for summarizing and synthesizing the growing body of research, providing clarity on current trends and identifying future directions [13]. This study incorporates an updated dataset of 6540 records, extending to 2025, to analyze the main topics, countries, institutions, authors, and journals in strabismus research. Our findings highlight both established and emerging research foci, offering recommendations for future investigation in the field.

Notably, a recent bibliometric study by Zhang et al. (2025) has also explored global research trends in strabismus using data from Web of Science [14]. Our work spans a longer period (1995-2025), uses a broader search strategy (TS=Title, Abstract, Keywords), and applies a fourth-degree polynomial model for productivity trend analysis. These differences allow us to present updated insights and uncover additional patterns in the field's evolution.

## Materials and Methods

**Figure-1:**
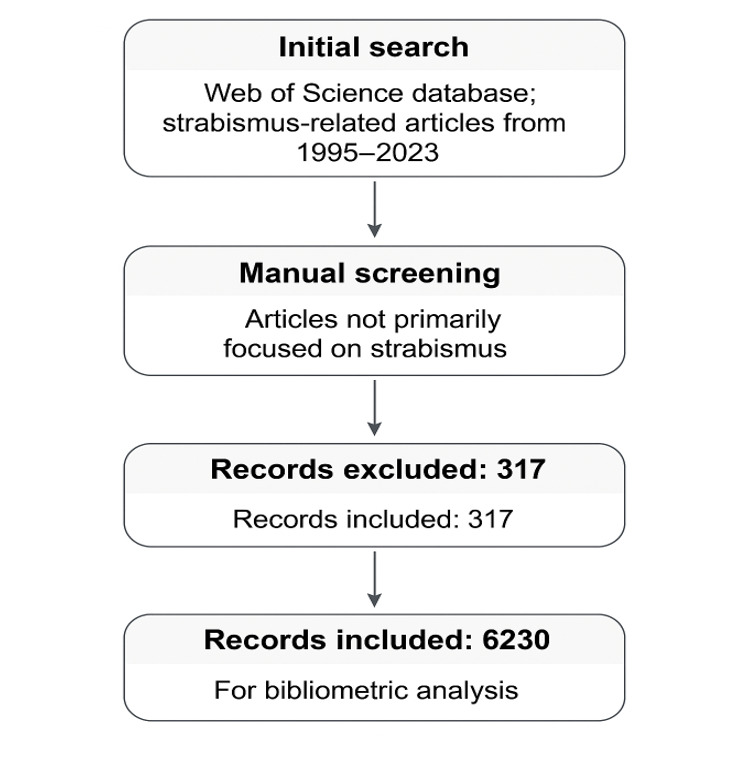
Figure 1. Flow chart of the data collection and analysis

**Figure-2:**
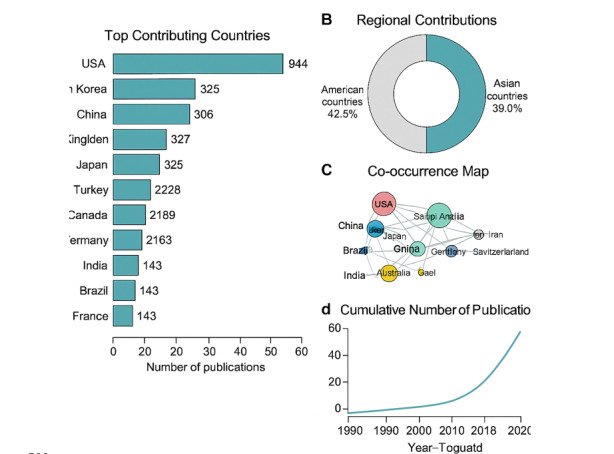
Figure 2. (a) The number of publications, citations, and H-index of the top 10 countries in the field of strabismus research.(b) The proportion of publications contributed by American, Asian, and other countries/regions. (c) The co-occurrence network of countries and regions based on collaborative publications. (d) Annual trends in the number of publications over the past 30 years and the corresponding contribution rates of the USA, South Korea, China, and other countries.

### Publication Search and Data Collection

A comprehensive literature search was conducted in the Web of Science (WOS) Core Collection, specifically the Science Citation Index Expanded (SCIE). The search covered the period from 1995 to 2025, and was performed using the topic field (TS), which includes the title, abstract, and author keywords. The search terms were as follows:

TS=("exotropia" OR "esotropia" OR "ocular deviation" OR "eye deviation" OR "squint" OR "strabismus" OR "hypertropia" OR "hypotropia" OR "heterotropia" OR "dissociated horizontal deviation" OR "dissociated vertical deviation" OR "dissociated torsional deviation")

A total of 6540 papers (articles and reviews) published in English from 1995 to 2025 were identified for manual screening. 317 papers that did not focus on strabismus as the main subject, such as articles on clinical trials related to pediatric surgical anesthesia management in strabismic children, or case reports on cranial trauma, cranial surgery, and systemic genetic syndromes in which strabismus was a concomitant symptom with little attention and no intervention, were manually excluded.

Finally, 6230 papers were included for further analysis (Figure-1). Data on publication year, countries or regions, citations, and authors of each relevant paper, as well as the H-index of each research team, were also acquired from WOS.

### Bibliometric Analysis

Data on publication contributions were analyzed using GraphPad Prism (v. 9.0.0.121), http://bibliometric.com/app, and Microsoft Excel 2016. To better fit the curve of cumulative publication numbers, the prediction model f(x)=ax^4 + bx^3 + cx^2 + dx + e was applied. The keyword co-occurrence network was visualized using VOSviewer (v. 1.6.20) [15] and CiteSpace (v. 6.1.R6) [16]. In the VOSviewer analysis, the occurrence threshold for exhibition was set to 11, while in the CiteSpace analysis, the time slice was set to 1 year, and the scale factor (k) was adjusted to 25. The selection of thresholds (e.g., minimum 10 publications for authors, minimum 30 occurrences for keywords) was based on prior bibliometric literature and adjusted to balance between visualization clarity and data comprehensiveness. Similar criteria have been used in other bibliometric studies employing VOSviewer to prevent overcrowded maps while preserving significant nodes for interpretation [17][18]. (van Eck & Waltman, 2010; Zyoud & Fuchs-Hanusch, 2020). These thresholds ensure that only the most influential items are included in the network analyses, improving interpretability.The dataset utilized for these analyses was updated to include 6540 records, reflecting the expanded search scope.

### Compliance with Ethics Guidelines

This bibliometric analysis was conducted based on a dataset comprising 6,540 previously published records and does not include any new studies involving human participants or animals performed by any of the authors.

## Results

**Figure-3:**
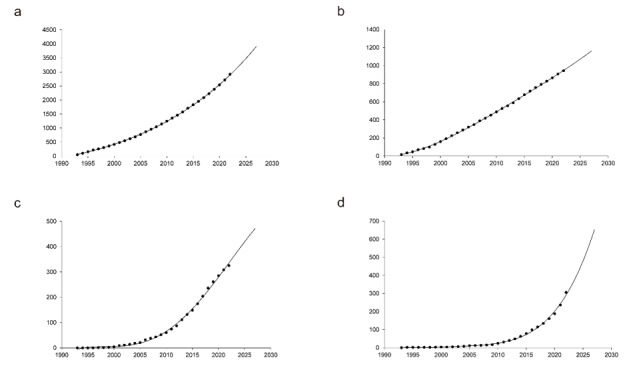
Figure 3. The growth trends of cumulative publication number over the recent 30 years and the matched curves, respectively. (a) Global. (b) The USA. (c) South Korea. (d) China

**Figure-4:**
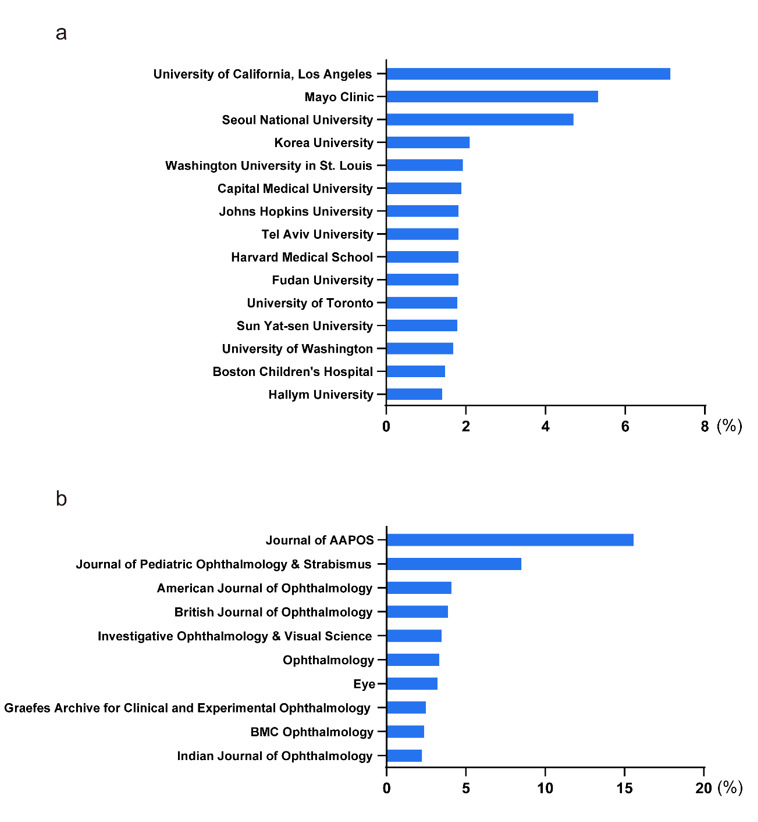
Figure 4. (a) Top 15 institutions with the largest publication amount on strabismus. (b) Top 10 journals with the most relevant publications

**Figure-5:**
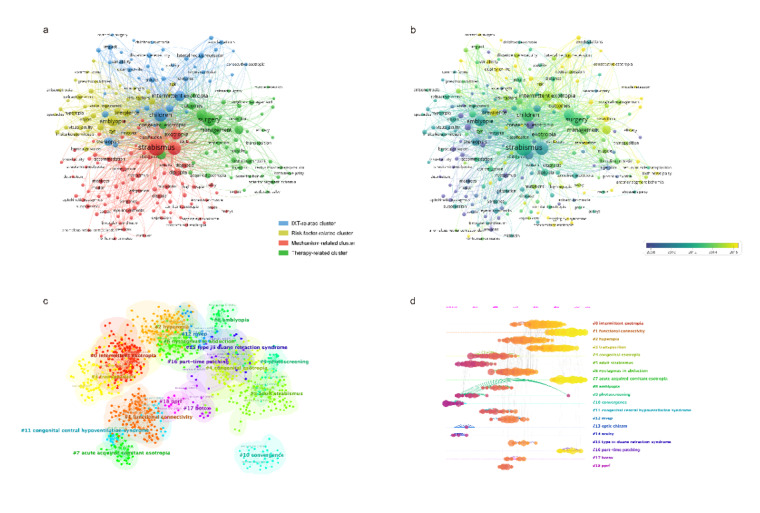
Figure 5. (a) Keywords in the strabismus field extracted via VOSviewer and clustered by co-occurrence. (b) Keywords extracted by VOSviewer and colored by the average time point of occurrence. (c) Keywords analyzed and clustered by CiteSpace with corresponding topic outlined for each cluster. (d) Figure 5c sorted by time zone

**Table:** Table1. Top 10 Publications in the Strabismus Field with the Most Citations

**Title**	**Corresponding authors**	**Journal**	**Publication year**	**Total citations**
Prevalence of amblyopia and strabismus in white and African American children aged 6 through 71 months: the Baltimore pediatric eye disease study	James M. Tielsch	Ophthalmology	2009	307
Incidence and types of childhood exotropia - A population-based study	Brian G. Mohney	Ophthalmology	2005	221
Prevalence and risk factors for common vision problems in children: data from the ALSPAC study	C. Williams	British Journal of Ophthalmology	2008	199
Amblyopia characterization, treatment, and prophylaxis	Kurt Simons	Survey of Ophthalmology	2005	198
Prevalence of amblyopia and strabismus in young Singaporean Chinese children	Audrey Chia	Investigative Ophthalmology & Visual Science	2010	166
Periventricular leukomalacia: an important cause of visual and ocular motility dysfunction in children	Gordon N. Dutton	Survey of Ophthalmology	2000	164
Instrument-induced measurement errors during strabismus surgery	Arthur L. Rosenbaum	Journal of AAPOS	1999	154
The effect of amblyopia on fine motor skills in children	Ann L. Webber	Investigative Ophthalmology & Visual Science	2008	149
The negative psychosocial impact of strabismus in adults	Angela N Buffenn	Journal of AAPOS	1999	147
The functional significance of stereopsis	Anna R. O’Connor	Investigative Ophthalmology & Visual Science	2010	144

**Table:** Table2. Top 10 Authors with the Most Publications on Strabismus

**Author**	**Country**	**Affiliation**	**Publication amount**	**Citation amount**
Jonathan M. Holmes	the USA	University of Arizona	68	844
Jeong-Min Hwang	South Korea	Seoul National University	65	439
Brian G. Mohney	the USA	Mayo Clinic	50	1136
Seung-Hyun Kim	South Korea	Korea University	42	184
Sarah R. Hatt	the USA	Mayo Clinic	41	629
Joseph L. Demer	the USA	University of California, Los Angeles	40	365
Hee Kyung Yang	South Korea	Seoul National University	40	170
Eileen E. Birch	the USA	University of Texas	39	655
David A. Leske	the USA	Mayo Clinic	39	557
Seong-Joon Kim	South Korea	Seoul National University	33	176

**Table:** Table3. Top 20 Burst Terms in the Strabismus Field in Recent 30 Years

**Keywords**	**Year**	**Strength**	**Begin**	**End**	**1993 - 2022**
Dissociated vertical deviation	2008	3.71	2020	2022	▂▂▂▂▂▂▂▂▂▂▂▂▂▂▂▂▂▂▂▂▂▂▂▂▂▂▂▃▃▃
prism adaptation	2012	4.2	2019	2020	▂▂▂▂▂▂▂▂▂▂▂▂▂▂▂▂▂▂▂▂▂▂▂▂▂▂▃▃▂▂
Sagging eye syndrome	2018	3.59	2018	2020	▂▂▂▂▂▂▂▂▂▂▂▂▂▂▂▂▂▂▂▂▂▂▂▂▂▃▃▃▂▂
recurrence	2017	3.98	2017	2022	▂▂▂▂▂▂▂▂▂▂▂▂▂▂▂▂▂▂▂▂▂▂▂▂▃▃▃▃▃▃
muscle transposition	2015	4.21	2015	2020	▂▂▂▂▂▂▂▂▂▂▂▂▂▂▂▂▂▂▂▂▂▂▃▃▃▃▃▃▂▂
consecutive exotropia	2010	4.38	2015	2019	▂▂▂▂▂▂▂▂▂▂▂▂▂▂▂▂▂▂▂▂▂▂▃▃▃▃▃▂▂▂
survival analysis	2014	6.17	2014	2020	▂▂▂▂▂▂▂▂▂▂▂▂▂▂▂▂▂▂▂▂▂▃▃▃▃▃▃▃▂▂
quality of life	2011	3.47	2014	2019	▂▂▂▂▂▂▂▂▂▂▂▂▂▂▂▂▂▂▂▂▂▃▃▃▃▃▃▂▂▂
surgical management	2004	6.68	2014	2017	▂▂▂▂▂▂▂▂▂▂▂▂▂▂▂▂▂▂▂▂▂▃▃▃▃▂▂▂▂▂
psychosocial aspect	2009	4.22	2011	2016	▂▂▂▂▂▂▂▂▂▂▂▂▂▂▂▂▂▂▃▃▃▃▃▃▂▂▂▂▂▂
refractive error	2005	5.4	2005	2011	▂▂▂▂▂▂▂▂▂▂▂▂▃▃▃▃▃▃▃▂▂▂▂▂▂▂▂▂▂▂
gene	2003	6.05	2003	2012	▂▂▂▂▂▂▂▂▂▂▃▃▃▃▃▃▃▃▃▃▂▂▂▂▂▂▂▂▂▂
heredity	2003	3.56	2003	2009	▂▂▂▂▂▂▂▂▂▂▃▃▃▃▃▃▃▂▂▂▂▂▂▂▂▂▂▂▂▂
early surgery	1996	10.12	1999	2009	▂▂▂▂▂▂▃▃▃▃▃▃▃▃▃▃▃▂▂▂▂▂▂▂▂▂▂▂▂▂
botulinum toxin A	1998	4.01	1998	2007	▂▂▂▂▂▃▃▃▃▃▃▃▃▃▃▂▂▂▂▂▂▂▂▂▂▂▂▂▂▂
adjustable suture	1997	4.1	1997	2004	▂▂▂▂▃▃▃▃▃▃▃▃▂▂▂▂▂▂▂▂▂▂▂▂▂▂▂▂▂▂
monkey	1995	5.81	1995	2007	▂▂▃▃▃▃▃▃▃▃▃▃▃▃▃▂▂▂▂▂▂▂▂▂▂▂▂▂▂▂
asymmetry	1994	4.73	1994	2001	▂▃▃▃▃▃▃▃▃▂▂▂▂▂▂▂▂▂▂▂▂▂▂▂▂▂▂▂▂▂
binocular vision	1994	6.63	1994	1997	▂▃▃▃▃▂▂▂▂▂▂▂▂▂▂▂▂▂▂▂▂▂▂▂▂▂▂▂▂▂
infantile esotropia	1993	8.08	1993	2004	▃▃▃▃▃▃▃▃▃▃▃▃▂▂▂▂▂▂▂▂▂▂▂▂▂▂▂▂▂▂

### Contributions and Co-occurrence of Nations and Regions

The number of publications related to strabismus has increased exponentially over the past 30 years (Figure-2d). Based on the updated dataset of 6,540 records, the United States remained the most prolific contributor, followed by South Korea and China (Figure-2a). The H-index of the USA continued to lead, reflecting its academic influence in this field. Approximately 83.8% of the papers originated from the top 10 most publishing countries, highlighting their academic centrality. Collectively, American countries and Asian countries contributed to 42.5% and 38.0% of the publications, respectively (Figure-2b). The USA maintained an annual output of over 40 publications in the past 15 years and remained the top contributor until 2020. In contrast, South Korea and China have demonstrated significant growth over the last decade, with China surpassing the USA in annual publication volume since 2021.

A co-occurrence analysis of 22 countries and regions (each with more than 20 publications) was conducted using VOSviewer[16] (Figure-2c). The countries were grouped into five clusters: (1) China, Japan, Saudi Arabia, Taiwan, Turkey, France, and Egypt; (2) the USA, Canada, Brazil, South Korea, and Iran; (3) Germany, Italy, Netherlands, Spain, and Switzerland; (4) England, India, Australia, and Israel; and (5) Sweden. Countries from the same continent often clustered together, which may reflect regional differences in strabismus epidemiology and management strategies [19].

### Publication Trends

The total number of publications on strabismus has demonstrated a consistent upward trajectory over the past three decades, and this trend is expected to continue in the coming years (Figure-3a). Based on the fitted predictive model, the cumulative number of publications is projected to exceed 8,000 within the next five years. The United States is expected to maintain its leading role in terms of publication volume, with a steady annual output contributing significantly to the global research landscape (Figure-3b-3d). In contrast, China has experienced a particularly rapid increase in publication output over the last five years. If this pace continues, China is projected to nearly double its cumulative publication count, potentially surpassing 600 papers by the end of the next five years (Figure-3d). This substantial growth highlights China's expanding influence and academic engagement in the field of strabismus research.

### Distribution of Citation

According to the WOS citation report, out of the 6540 relevant records on strabismus publications since 1995, 26,730 non-self-citations were identified. Each publication has been cited an average of 7.07 times. The United States contributed the most citations (18,650 citations, including 15,500 non-self-citations) and has the highest H-index (54) over the past three decades. Interestingly, England ranks second in both H-index (28) and non-self-citations (4,350), despite being fourth in overall publication volume, approximately half that of South Korea and China. This phenomenon may relate to England's early involvement in strabismus research. South Korea ranks third, with 2,924 citations, 2,327 non-self-citations, and an H-index of 24.

The paper with the highest citation count has received 307 citations, while the least-cited ones have not yet received any citations. All relevant papers were divided into three groups based on citation frequency: high citation frequency group (more than 200 citations), medium citation frequency group (more than 100 but no more than 200 citations), and low citation frequency group (no more than 100 citations). The vast majority of publications were in the low citation frequency group, while 19 papers were in the medium citation frequency group and only 2 papers were in the high citation frequency group. "Prevalence of Amblyopia and Strabismus in White and African American Children Aged 6 through 71 Months: The Baltimore Pediatric Eye Disease Study" is the most cited paper at present, with David S. Friedman as the corresponding author [19]. Notably, both of the papers in the high citation frequency group were published in the authoritative and classic journal, Ophthalmology [20][21]. The top 10 most frequently cited publications are displayed in Table-1. The main findings of these relatively more frequently cited publications relate to the prevalence, types, and associated factors of strabismus derived from retrospective studies with large sample sizes and long durations.

We generated heatmaps to understand the distribution of citations across years for each group (Figures-S2a and -S2b). In these two heatmaps, each row represents a paper, and each column represents a year, respectively. The color density indicates citation frequency, as described in the corresponding legends. Additionally, we analyzed the publication year of each paper in different groups (Figure-S2c). It is clear that the articles in the high and medium citation frequency groups, most of which were published around the 2000s, were seminal in the development of strabismus research.

### Contributions of Institutions

Over the past 30 years, the University of California, Los Angeles has been the leading institution with 460 publications (7.03%) in the field of strabismus research, followed by Mayo Clinic with 348 publications (5.30%) and Seoul National University with 314 publications (4.79%). The top 15 institutions are listed in (Figure-4a).

### Distribution in Journals

Nearly half of the relevant publications (3210, 49.08%) were published in the top 10 periodicals. Journal of AAPOS led with 1016 articles (15.53%) and is arguably the most authoritative journal in the field of strabismus. Journal of Pediatric Ophthalmology & Strabismus ranked second with 538 publications (8.23%), followed by American Journal of Ophthalmology with 256 articles (3.92%) (Figure-4b).

In 2014, a high-quality article—currently the one with the highest impact factor among relevant papers—authored by Shilpa Gulati et al. was published in JAMA Pediatrics. It was a longitudinal cohort study involving 38,055 otherwise healthy premature infants. The study found that very low birth weight significantly increased the risk of strabismus. The authors thus advocated for updated clinical guidelines to enhance health surveillance in preterm infants [22].

Moreover, the high-impact journal Survey of Ophthalmology published only 14 relevant articles, yet two of them were among the top 10 most-cited papers discussed earlier [23][24]. This indicates the influential role of the journal itself in driving citation impact.

### Contributions and Co-occurrence of Authors

In the past three decades, 457 papers (6.99%) were published by the top 10 authors in the field of strabismus. Jonathan M. Holmes from University of Arizona published the most, with 68 papers (40 as the corresponding author and 13 as the first author) and 844 citations. Works of Jeong-Min Hwang from Seoul National University were published the second most, with 65 papers (52 as the corresponding author and 3 as the first author) and 439 citations. Brian G. Mohney from Mayo Clinic ranked third with 50 papers (29 as the corresponding author and 10 as the first author) and 1136 citations. Prof. Mohney is also the most impactful author (with the highest total citation count) and was a co-author of the second most cited paper, Incidence and Types of Childhood Exotropia: A Population-Based Study[2]. All top 10 authors are from the USA or South Korea; 3 of them are affiliated to Mayo Clinic and 3 are affiliated to Seoul National University (Table-2).

The collaboration between researchers was also analyzed using VOSviewer (Figure-S3). Node size represents the contribution of the investigators individually, while line thickness indicates the correlation strength between the connected authors. According to the analysis, Jonathan M. Holmes cooperate quite closely with others. 3 of the top 10 authors from Mayo Clinic, as discussed above (Table-2), exhibited a close connection (Figure-S3, lower right), so did the 3 scholars from Seoul National University (Figure-S3, lower left corner). Clinical studies with large sample sizes often require collaboration across groups and institutions, and closer communication can also lead to more meaningful results with more generalizable implications.

### Co-occurrence Analysis of Keywords and the Burst Terms

Keyword analysis reveals the words that are used most frequently within the field of strabismus and how they relate to each other, giving a clue for emerging trends and hotspots. We analyzed noun words with more than 11 occurrences in these 6540 relevant papers using VOSviewer. After merging the duplicates and excluding irrelevant words, we obtain 218 keywords. These words can be roughly classified into four clusters based on co-occurrence (Figure-5a). Except for the blue cluster, which is specifically related to intermittent exotropia (IXT), the remaining three can be summarized as risk factor-related cluster (yellow), mechanism-related cluster (red), and therapy-related cluster (green).

We colored the nodes based on the average time point of occurrence (Figure-5b). Yellowish words (e.g., quality-of-life, consecutive esotropia and sagging eye syndrome), appeared more recently, while larger and darker green nodes, such as surgery, children and amblyopia might be the topic of constant interests in the strabismus community.

Publications were also analyzed by CiteSpace [25] with a time slice of 1 year (Figure-5c and -5d). The modularity Q was 0.795, reflecting the significance of the network, and the weighted mean silhouette S was 0.8611, which means that the clusters are reasonable. In these two maps, each node represents a keyword while each line (both the colored and the gray ones) for the co-occurrence relationship, respectively. 1248 unique nodes, 4763 lines were contained, and 19 main clusters were produced (Figure-5c).

In the map sorted by time zone (Figure-5d), the horizontal position of each node represents the initial the corresponding keywords co-occurred, while the size reflects the frequency. As shown, IXT, functional connectivity and hyperopia are highly topical, with a large bunch of relevant keywords, while IXT, acute acquired comitant esotropia (AACE) and part-time patching are at the frontier of current research in the last 10 years.

The burst terms were outlined by CiteSpace to understand the dynamic transformations in research focus and current hotspots, so as to predict subsequent trends in the future. After the screening of burst strength, 20 burst terms were listed (Table-3), along with the year of the first appearance and the specific time span of burstiness. The last column is a visualization of the corresponding time information. As we can see, the treatment of strabismus, especially surgical treatment, has been a long-standing topic throughout recent 30 years. In the earlier period, the pathogenesis of strabismus (binocular vision, heredity and gene etc.) received great attention, while more recently, research interests in postoperative management as well as complex types of strabismus (dissociated vertical deviation, sagging eye syndrome, and recurrence etc.) burst significantly. These results are also consistent with those from VOSviewer (Figure-5b).

## Discussion

Our bibliometric analysis provides insight into the evolving frontiers and future directions of strabismus research. By analyzing high-frequency keywords and recent burst terms from multiple perspectives—such as disease subtype, underlying mechanisms, diagnostic innovations, and treatment strategies—we identified several prominent themes. These include intermittent exotropia (IXT), acute acquired comitant esotropia (AACE), pediatric populations, stereopsis, functional magnetic resonance imaging (fMRI), surgical outcomes, and various non-surgical or complementary interventions. Collectively, these findings offer a comprehensive overview of global progress in the field over the past three decades (1995-2025) and may help forecast future research priorities.

The annual number of publications on strabismus has grown exponentially (Figure-2d), reflecting the expanding global interest in this domain. The United States and Asian countries, particularly China and South Korea, have been major contributors (Figure-2b). The USA remains the most influential country, with the highest number of publications (944, 32.4%), total citations (16,180), and H-index (54) (Figure-2a). This leadership likely stems from its robust research infrastructure, higher reported prevalence of strabismus [26], early academic engagement in pediatric ophthalmology, and the presence of high-impact journals—several of which rank among the top three in strabismus research output (Figure-4b). These factors collectively reinforce the dominant position of the United States over the past 30 years.

South Korea ranks second in publication output and has leveraged its advanced cosmetic and ophthalmic industries to drive research forward. The demand for aesthetically favorable outcomes in strabismus patients, particularly in younger populations, may partly explain South Korea’s regional prominence in the field. Notably, China has demonstrated the most rapid growth in publication volume. Although its contributions remained under 100 papers until 2017, it nearly caught up with South Korea within just five years (Figure-3), underscoring a swift and strategic expansion in academic engagement.

It is logical that the co-occurrence analysis of countries and regions revealed stronger connections between countries from the same continent (Figure-1). Previous epidemiological studies have indicated regional variations in the spectrum of strabismus. Specifically, exotropia is more commonly reported in Asian children, whereas esotropia is less frequent compared to children in Western countries [21][27][28]. The dose-effect relationship of strabismus surgery may also vary among different ethnic groups based on clinical experience.

In our study, we identified the top journals publishing research on strabismus. Interestingly, the Journal of AAPOS has a relatively low impact factor, but it holds the largest number of relevant publications (454, 15.6%) (Figure-4b) and has the highest citation count (3316). The Journal of AAPOS is the official journal of the American Association for Pediatric Ophthalmology and Strabismus (AAPOS), which plays a crucial and authoritative role in the field of strabismus. It is not uncommon for an ophthalmology journal to have a low impact factor but still be considered highly influential in its specialty.

Citation counts and H-indexes provide insights into the influence of specific research teams [29][30]. Highly cited articles, authors, and institutions often lead future research directions. Despite the rapid growth in publication quantity, it is concerning that China, despite its significant publication output, lags behind countries like England in citation counts and H-index. China’s top institution in strabismus research, Fudan University, ranks tied for seventh globally with 53 publications, but its citation count is much lower compared to institutions ranked similarly. Moreover, the Chinese journal with the most publications, International Journal of Ophthalmology, ranks 19th globally. This could be attributed to the earlier focus on quantity over quality in Chinese hospitals and the underdevelopment of healthcare record systems. However, with the growing emphasis on research quality and improvements in medical environments, we can expect more high-quality studies from China in the near future.

Among all the identified keywords, intermittent exotropia (IXT) has been the most frequently discussed over the years (Figure-5), largely due to its high prevalence. A report from the United States indicated that the prevalence of IXT was 0.86% in children under 11 years old [21], while a Chinese study found a prevalence of 4.5% in children aged 36-72 months [27]. The management of IXT has consistently been a major research focus. Currently, surgery is the primary recommendation for restoring ocular alignment in patients with large-angle or frequently observed IXT [31]. Notably, nearly half of the hotspots in strabismus research are related to treatments, particularly the surgical management of strabismus (Table 3). Keywords such as surgical outcomes, recurrence, consecutive strabismus, and muscle transposition emerged around 2017, reflecting an increasing interest in the outcomes of surgical interventions [12][32][33] and the ongoing pursuit of more precise surgical techniques.

Bilateral lateral rectus recession (BLR) and unilateral recession-resection (R&R) procedures, both fundamental surgical options for IXT, were proposed around fifty years ago. Recent studies have expanded on their dose-response relationship, surgical risks, and countermeasures [34]. However, these findings have been controversial due to variations in population types, strabismus subtypes (e.g., basic or divergence excess), and follow-up periods. Some randomized controlled trials (RCTs) have demonstrated that the BLR procedure offers more stable therapeutic effects over longer observation periods [35][36], while others have found that the R&R procedure yields a higher short-term success rate [8][37]. Nevertheless, surgical designs strictly based on formulae may still lead to recurrence or overcorrection, due to individual variability and the drift phenomenon. After one year of follow-up, the success rate ranges from 42% to 74.2% [36][38], while the overcorrection rate ranges from 1.5% [38] to 21% [39]. Additional interventions, including secondary surgery, may be required for patients with suboptimal alignment.

Additionally, non-surgical strategies such as botulinum toxin A injection, patching, binocular single vision training, and glasses are continually reshaping the landscape of strabismus interventions [12] (Figure-5d). Botulinum toxin A injection is a less invasive treatment with shorter-lasting side effects, and has been reported to be similarly effective to surgery, especially for small-angle strabismus [40][41]. It can restore the parallelism of visual axes and binocular single vision, providing an opportunity for the central nervous system to regain control of eye position [42]. However, many clinical ophthalmologists remain cautious about this therapy due to concerns such as the lack of long-term data and some uncertainty regarding dosage. Preoperative and postoperative binocular single vision training, along with patching (occlusion of the dominant eye or alternate occlusion in children with no dominant eye identified [43]), may assist in improving the surgical success rate [10]. The development of handheld smart devices has also made vision training more convenient. Overall, there is a need for more extensive and long-term research on surgical outcomes to optimize the timing, choice of procedures, and postoperative management for intermittent exotropia (IXT) and other complex forms of strabismus. The refinement of emerging therapies is also anticipated.

In recent discussions, the term "functional connectivity" has gained prominence (Figure-5d), which refers to the functional integration between brain regions in resting-state fMRI [44]. Disrupted brain networks lead to abnormalities in binocular vision and the oculomotor system, which is believed to be one of the fundamental mechanisms for comitant strabismus [3]. Rapid advancements in fMRI technology have offered new insights into the etiology of strabismus related to stereopsis and the central nervous system. Unlike conventional MRI, which provides images of orbital and cranial structures, as well as the thickness, starting, and ending points of extraocular muscles, fMRI reveals how different brain regions function and interact. The dorsal visual pathway is often reported to be impaired in strabismus patients according to fMRI [7][45]. However, the changes observed in different studies are not always consistent, due to the significant heterogeneity of the subjects [46]. Nishida Y. et al. suggested that regions from the dorsal portion of the occipital lobe to the superior parietal lobule are responsible for processing stereopsis [47], while Hu Y. et al. identified functional changes in the fusiform gyrus related to the deviation angle in AACE patients [7]. fMRI also offers an objective tool for predicting stereopsis recovery after strabismus surgery. Xi S. et al. demonstrated a correlation between worse postoperative stereopsis and hypoactivity in the right V3A and left intraparietal sulcus in patients with IXT [48]. In conclusion, although further clinical practice is necessary to establish standardized guidelines, fMRI holds promise as a valuable tool for studying mechanisms, prognostic assessments, and postoperative management of strabismus. This may represent an important direction for future research.

Recently, another bibliometric analysis of strabismus research was published by Zhang et al. in Frontiers in Medicine (2025), which analyzed 4,517 records retrieved from the Web of Science database using TS (Title, Abstract, and Keywords) strategy and VOSviewer. While both studies aimed to map the scientific landscape of strabismus research, their approaches and conclusions differ. Compared to their narrower time coverage (2000-2023), our study includes a broader timespan (1995-2025) and provides a more comprehensive trend analysis with updated data. Furthermore, we employed a fourth-degree polynomial model for author productivity, allowing better curve fitting to the data’s nonlinear growth, while their study relied on more traditional Lotka’s law assumptions. These methodological differences underscore the complementary nature of both analyses, offering a richer understanding of the field's development from distinct angles.

While Zhang et al.'s study provided useful visualizations and identified leading countries and institutions, it did not assess productivity models in-depth, nor did it evaluate author-level metrics using polynomial trend analysis. In contrast, our study offers detailed insights into productivity distributions, keyword evolution, and citation dynamics. Therefore, this work complements existing bibliometric analyses and enriches the understanding of research trends in strabismus.

Efforts to explore the mechanisms of strabismus have extended beyond fMRI studies of patients. Several etiologically relevant keywords, such as "gene," "hereditary," and "refractive error," have emerged. Attention to hereditary and refractive errors started around 20 years ago and surged over the last decade (Figure-5b). Apart from systemic muscle disorders and abnormalities in the development of extraocular muscles, for which clear causative genes have been identified (e.g., DMD for Duchenne muscular dystrophy [49], KIF21A for congenital fibrosis of the extra.

The use of a fourth-degree polynomial model to analyze author productivity represents a methodological strength of this study. Unlike traditional approaches such as Lotka’s law or linear regressions, which may oversimplify the data, our approach captured the dynamic and nonlinear evolution of productivity over three decades. This enhanced model supports a more nuanced understanding of author contribution patterns in the strabismus literature.

## Conclusion

Overall, this is one of the first bibliometric analyses of strabismus publications that examines the distribution of country, institution, author, journal, and citations over the past three decades and forecasts future publication trends. We identified the pivotal role of the United States in this field and found that high-quality prospective clinical studies with extensive participant involvement and extended observation periods on high-prevalence types of strabismus were more popular in authoritative journals. These studies could help resolve currently controversial issues and better meet the clinical needs in strabismus practice. We also summarized the transformation of research focus in the field of strabismus. While IXT, risk factors, and treatments for strabismus have been longstanding hot topics, in recent years, concerns about AACE, complex types of strabismus, and long-term quality of life have continued to grow. These results may help relevant researchers and clinicians develop a general understanding of the history of the strabismus field and make better decisions regarding future research.

## Conflict of Interest

All authors (Minglian Ye, Jianzhong Yang, Jiamei Luo) declare that they have no conflicts of interest and no collaboration with the authors of the recently published study in Frontiers in Medicine (DOI: 10.3389/fmed.2025.1488817).

## References

[R1] Hassan MB, Hodge DO, Mohney BG (2015). Prevalence of Mental Health Illness among Patients with
Adult-Onset Strabismus. Strabismus.

[R2] Graham PA (1974). Epidemiology of strabismus. Br J Ophthalmol.

[R3] Bui Quoc, Milleret C (2014). Origins of strabismus and loss of binocular vision. Frontiers in integrative neuroscience.

[R4] Cotter SA, Varma R, Tarczy-Hornoch K (2011). Risk factors associated with childhood strabismus: the
multi-ethnic pediatric eye disease and Baltimore pediatric eye disease
studies. Ophthalmology.

[R5] Donnelly UM (2012). Horizontal strabismus worldwide--what are the risk factors. Ophthalmic epidemiology.

[R6] Tychsen L (2007). Causing and curing infantile esotropia in primates: the role of
decorrelated binocular input (an American Ophthalmological Society thesis). Transactions of the American Ophthalmological Society.

[R7] Hu Y, Wang S, Wu L, Xi S, Wen W, Zhao C (2023). Deficits of Visual Cortex Function in Acute Acquired Concomitant
Esotropia Patients. Invest Ophthalmol Vis Sci.

[R8] Choi J, Chang JW, Kim SJ, Yu YS (2012). The long-term survival analysis of bilateral lateral rectus
recession versus unilateral recession-resection for intermittent exotropia. American journal of ophthalmology.

[R9] Lee S, Lee YC (2001). Relationship between motor alignment at postoperative day 1 and
at year 1 after symmetric and asymmetric surgery in intermittent exotropia. Japanese journal of ophthalmology.

[R10] Figueira EC, Hing S (2006). Intermittent exotropia: comparison of treatments. Clinical & experimental ophthalmology.

[R11] Sharma P, Gaur N, Phuljhele S, Saxena R (2017). What's new for us in strabismus. Indian journal of ophthalmology.

[R12] Joyce KE, Beyer F, Thomson RG, Clarke MP (2015). A systematic review of the effectiveness of treatments in
altering the natural history of intermittent exotropia. Br J Ophthalmol.

[R13] Casado-Aranda LA, Sánchez-Fernández J, Viedma-Del-Jesús MI (2021). Analysis of the scientific production of the effect of COVID-19
on the environment: A bibliometric study. Environmental research.

[R14] Xiu Y, Zhang Y, Su Y, Zhu C, Liu Z (2025). A bibliometric analysis of strabismus (from 2004 to 2023). Frontiers in Medicine.

[R15] van Eck, Waltman L (2010). Software survey: VOSviewer, a computer program for bibliometric
mapping. Scientometrics.

[R16] Chen C (2004). Searching for intellectual turning points: progressive knowledge
domain visualization. Proceedings of the National Academy of Sciences of the United States of
America.

[R17] Van Eck, Waltman L (2010). Software survey: VOSviewer, a computer program for bibliometric
mapping. scientometrics.

[R18] Zyoud S, Al-Jabi S, Sweileh W (2014). Bibliometric analysis of scientific publications on waterpipe
(narghile, shisha, hookah) tobacco smoking during the period 2003-2012. Tobacco Induced Diseases.

[R19] Yu X, Ji Z, Yu H, Xu M, Xu J (2016). Exotropia Is the Main Pattern of Childhood Strabismus Surgery in
the South of China: A Six-Year Clinical Review. J Ophthalmol.

[R20] Friedman DS, Repka MX, Katz J (2009). Prevalence of amblyopia and strabismus in white and African
American children aged 6 through 71 months the Baltimore Pediatric Eye
Disease Study. Ophthalmology.

[R21] Govindan M, Mohney BG, Diehl NN, Burke JP (2005). Incidence and types of childhood exotropia: a population-based
study. Ophthalmology.

[R22] Gulati S, Andrews CA, Apkarian AO, Musch DC, Lee PP, Stein JD (2014). Effect of gestational age and birth weight on the risk of
strabismus among premature infants. JAMA pediatrics.

[R23] Simons K (2005). Amblyopia characterization, treatment, and prophylaxis. Surv Ophthalmol.

[R24] Jacobson LK, Dutton GN (2000). Periventricular leukomalacia: an important cause of visual and
ocular motility dysfunction in children. Surv Ophthalmol.

[R25] Chen C, Chen Y (2005). Searching for clinical evidence in CiteSpace. AMIA Annual Symposium proceedings AMIA Symposium.

[R26] Buffenn AN (2021). The impact of strabismus on psychosocial heath and quality of
life: a systematic review. Surv Ophthalmol.

[R27] Chen X, Fu Z, Yu J, et al (2016). Prevalence of amblyopia and strabismus in Eastern China: results
from screening of preschool children aged 36-72 months. Br J Ophthalmol.

[R28] Greenberg AE, Mohney BG, Diehl NN, Burke JP (2007). Incidence and types of childhood esotropia: a population-based
study. Ophthalmology.

[R29] Bertran K, Cortey M, Díaz I (2020). The use of H-index to assess research priorities in poultry
diseases. Poultry science.

[R30] Nowak JK, Lubarski K, Kowalik LM, Walkowiak J (2018). H-index in medicine is driven by original research. Croatian medical journal.

[R31] Buck D, Hatt SR, Haggerty H (2007). The use of the Newcastle Control Score in the management of
intermittent exotropia. Br J Ophthalmol.

[R32] Sun Y, Zhang T, Chen J (2018). Bilateral lateral rectus recession versus unilateral recession
resection for basic intermittent exotropia: a meta-analysis. Graefes Arch Clin Exp Ophthalmol.

[R33] Pang Y, Gnanaraj L, Gayleard J, Han G, Hatt SR (2021). Interventions for intermittent exotropia. Cochrane Database Syst Rev.

[R34] Shen T, Kang Y, Lin X, Wu H, Yan J (2022). Newly developed abnormal head position and secondary esotropia
after strabismus surgery for children with intermittent exotropia. International ophthalmology.

[R35] Kushner BJ (1998). Selective surgery for intermittent exotropia based on
distance/near differences. Archives of ophthalmology (Chicago, Ill : 1960).

[R36] Chia A, Seenyen L, Long QB (2006). Surgical experiences with two-muscle surgery for the treatment of
intermittent exotropia. Journal of AAPOS : the official publication of the American Association
for Pediatric Ophthalmology and Strabismus.

[R37] Maruo T, Kubota N, Sakaue T, Usui C (2001). Intermittent exotropia surgery in children: long term outcome
regarding changes in binocular alignment A study of 666 cases. Binocular vision & strabismus quarterly.

[R38] Buck D, Powell CJ, Rahi J (2012). The improving outcomes in intermittent exotropia study: outcomes
at 2 years after diagnosis in an observational cohort. BMC ophthalmology.

[R39] Lee SY, Hyun Kim, Thacker NM (2007). Augmented bilateral lateral rectus recessions in basic
intermittent exotropia. Journal of AAPOS : the official publication of the American Association
for Pediatric Ophthalmology and Strabismus.

[R40] Li Y, Wu X (2008). [Observation of botulinum toxin A management in childhood with
intermittent exotropia]. [Zhonghua yan ke za zhi] Chinese journal of ophthalmology.

[R41] Spencer RF, Tucker MG, Choi RY, McNeer KW (1997). Botulinum toxin management of childhood intermittent exotropia. Ophthalmology.

[R42] Ripley L, Rowe FJ (2007). Use of botulinum toxin in small-angle heterotropia and
decompensating heterophoria: a review of the literature. Strabismus.

[R43] Buck D, Powell C, Cumberland P (2009). Presenting features and early management of childhood
intermittent exotropia in the UK: inception cohort study. Br J Ophthalmol.

[R44] Lv H, Wang Z, Tong E (2018). Resting-State Functional MRI: Everything That Nonexperts Have
Always Wanted to Know. AJNR American journal of neuroradiology.

[R45] Yan X, Lin X, Wang Q (2010). Dorsal visual pathway changes in patients with comitant
extropia. PloS one.

[R46] Guo W, Zhu H, Xu XQ, Hu H, Liu H (2023). [Research progress of brain MRI in comitant strabismus]. [Zhonghua yan ke za zhi] Chinese journal of ophthalmology.

[R47] Nishida Y, Hayashi O, Iwami T (2001). Stereopsis-processing regions in the human parieto-occipital
cortex. Neuroreport.

[R48] Xi S, Zhou Y, Yao J (2023). Cortical Deficits are Correlated with Impaired Stereopsis in
Patients with Strabismus. Neurosci Bull.

[R49] Shieh PB (2013). Muscular dystrophies and other genetic myopathies. Neurologic clinics.

